# Flupyradifurone, imidacloprid and clothianidin disrupt the auditory processing in the locust CNS

**DOI:** 10.1007/s00359-025-01735-8

**Published:** 2025-02-13

**Authors:** Marcelo Christian, Michelle Kraft, Paul Wilknitz, Manuela Nowotny, Stefan Schöneich

**Affiliations:** Institute for Zoology and Evolutionary Research, Friedrich Schiller University Jena, Erbertstraße 1, 07743 Jena, Germany

**Keywords:** Insect hearing, Auditory processing, Neonicotinoid insecticides, Flupyradifurone, Imidacloprid, Clothianidin

## Abstract

Since the EU banned classic neonicotinoids like imidacloprid and clothianidin, they may be replaced by more recently marketed insecticides such as flupyradifurone. However, they all operate on the same neuropharmacological principle as selective agonists at the insect’s nicotinic acetylcholine receptors. Here we investigated the impact of flupyradifurone, imidacloprid and clothianidin on the neuronal processing in the auditory pathway of the desert locust *Schistocerca gregaria*. While stepwise increasing the insecticide concentration in the haemolymph, we extracellularly recorded the spike responses of auditory afferents in the tympanal nerve and of auditory interneurons in the neck connectives. All three insecticides showed a very similar dose-dependent suppression of spike responses in the auditory interneurons ascending towards the brain, whereas the spike responses in the sensory neurons of the ears appeared unaffected. Furthermore, by systematic injection experiments we demonstrate that insecticide dosages which already supress the information transfer in the auditory pathway are by far too low to induce the typical poisoning symptoms like trembling, spasms, and paralysis. We discuss how sublethal intoxication with classical neonicotinoids or functionally related insecticides like flupyradifurone may disrupt the postsynaptic balance between excitation and inhibition in the auditory pathway of locusts and other orthopteran insects.

**Supplementary Information:**

The online version contains supplementary material available at 10.1007/s00359-025-01735-8.

## Introduction

The use of insecticides in the industrialized agricultural sector is a crucial tool to prevent crop shortfall or harvest failure. Since their market introduction in the 1990s, neonicotinoids quickly became the most widely used class of insecticides in the world (Simon-Delso et al. 2015; Thompson et al. 2020). The major advantage of this pesticide type is that these neuroactive chemicals selectively target insects that feed on crops, while being much less harmful to vertebrates, including human consumers (Matsuda et al. 2001). However, the worldwide soaring agricultural use of neonicotinoid insecticides over the last decades is suspected to be one significant factor contributing to the global decline of insect populations and biodiversity (Pisa et al. 2017; Hallmann et al. 2017). To better protect pollinators and other non-target insects, the outdoor use of some classical neonicotinoids (including imidacloprid and clothianidin) has been banned in the EU since 2018 (Sgolastra et al. 2020). The agricultural industry, however, may simply replace them by more recently marketed substitutes like flupyradifurone (Nauen et al. 2015; Thompson et al. 2020). This neonicotinoid-like insecticide had been developed to overcome emerging resistance of some pest insects to classical neonicotinoids and has also been marketed as safer for pollinating bees (Bass et al. 2015; Campbell et al. 2016; Matsuda et al. 2020). Similar to the nitroimines imidacloprid and clothianidin, the butenolide flupyradifurone is a cholinergic agonist that was synthetically designed to selectively bind to the nicotinic acetylcholine-receptors (nAChR) in the insect central nervous system (CNS) (Nauen et al. 2015; Crossthwaite et al. 2017; Taillebois et al. 2018) and therefore has a much higher potency in insects than in mammals and other vertebrates (Casida 2018; Matsuda et al. 2020).

The nAChRs in the insect CNS are ligand-gated cation channels that primarily evoke excitatory postsynaptic depolarizations in the dendrites of interneurons when activated by presynaptic release of the neurotransmitter acetylcholine into the synaptic cleft. Therefore, neonicotinoids intoxication usually causes long-lasting membrane depolarization and consequently a lower threshold for spike generation in interneurons with postsynaptic nAChRs (Buckingham et al. 1997). Since acetylcholine is considered to be the major excitatory neurotransmitter in the insect nervous system (Homberg 2002), an agonist-induced overactivation of nAChRs may also impact the balance between excitation and inhibition in central neural networks. The most common symptoms of increasing neonicotinoid intoxication in insects include sensory and cognitive impairment, hyperactivity, trembling, muscle cramps, spasms, and finally complete paralysis, which can eventually lead to death (Tharp et al. 2000; Suchail et al. 2001; Scheibli et al. 2023). While this is the intended effect on pest insects when they feed on neonicotinoid treated crops in the field, contamination of adjacent habitats and groundwater with these insecticides can also severely impact on the fitness in a wide range of non-target invertebrates (Pisa et al. 2017).

Due to the direct economic implications of a decline in pollinating insects (Gallai et al. 2009), most research studies initially focused on the impact of neonicotinoids on bees and bumblebees (Lu et al. 2020) to provide a scientific basis for debates on regulatory measures (Sgolastra et al. 2020). When exposed to sublethal dosages of classic neonicotinoids like imidacloprid or clothianidin, bees show a complex spectrum of impairments in their basic motor functions (Williamson et al. 2014), olfactory memory and learning capacity (Williamson and Wright 2013), which all negatively impact on the navigation and orientation skills required for foraging (Yang et al. 2008; Fischer et al. 2014) and their social behaviour (Crall et al. 2018). Consequently, neonicotinoid contamination can drastically lower the colony performance and therefore increase the risk of colony collapse (Lu et al. 2014, 2020). In recent studies, flupyradifurone was reported to negatively impact memory, cognition, foraging behaviour, and survival in bees (Hesselbach and Scheiner 2018; Hesselbach et al. 2019; Tosi et al. 2021). It further affects immune responses and the gut microbiome, especially when bees are exposed to a combination of pesticides and pathogens (Harwood et al. 2022; Al Naggar et al. 2022). Besides a large body of accumulating evidence regarding the risks for bees, a growing number of studies is now focusing on the impact of neonicotinoids and related insecticides on other non-target insects with very different lifestyles, e.g. ants (Schlaeppi et al. 2020; Frizzi et al. 2023), moths (Navarro-Roldán and Gemeno 2017), wasps (Tappert et al. 2017), lacewings and lady beetles (Scheibli et al. 2023, 2024). However, nothing is known yet on how these types of insecticides affect the hearing system in any insect species. Therefore, here we investigated the effects of flupyradifurone and the two classical neonicotinoids imidacloprid and clothianidin on the neuronal processing in the auditory pathway in the CNS of desert locusts *Schistocerca gregaria*. Locusts are among the most devastating pests worldwide when they form swarms in plague years, which then can cause devastating damages to crop production (Peng et al. 2020). In this study, however, we also see the locusts as a well-studied neurobiological model (Burrows 1996) representing the huge group of orthopteran insects with tympanic ears for acoustic communication and/or predator avoidance (Stumpner and von Helversen 2001; Schöneich 2020).

The tympanic ears of the locust are situated laterally at the tergite of the first abdominal segment, one on each body side (Gray 1960). Attached to a tympanic membrane, the auditory ganglion (Müller’s organ) houses the somata of 60–80 auditory receptor cells (Jacobs et al. 1999). The axons of the sensory neurons enter the fused metathoracic ganglion complex (T3-A3) via the auditory nerve (N6), which is easily accessible for extracellular recordings (Warren et al. 2020). In the thoracic ganglia the axonal terminals of auditory afferents provide the synaptic input to 10–15 excitatory and inhibitory local interneurons. These first-order auditory interneurons then drive and shape the spike responses of 15–20 interneurons with ascending axons that forward auditory information through the neck connectives towards the brain (Kalmring 1975; Römer and Marquart 1984; Wirtssohn and Ronacher 2015). The auditory pathway in the thoracic ganglia of locusts has been studied intensively at the neuronal level over the last decades and can be summarized as a hierarchically organized feedforward network with strong excitatory and inhibitory synaptic connections between consecutive processing levels (Vogel and Ronacher 2007; Eberhard et al. 2014). Accumulating evidence suggest that acetylcholine is most likely the excitatory neurotransmitter of the sensory afferents (Homberg 2002), whereas many local auditory interneurons in the thoracic ganglia inhibit other local and/or intersegmental projecting interneurons (Stumpner and von Helversen 2001). To test the impact of flupyradifurone, imidacloprid and clothianidin on the neuronal network for auditory processing, we extracellularly recorded in more than 50 locusts simultaneously the spike responses of auditory afferents as sum potentials in the tympanal nerve (T3-N6) and the individual action potentials of ascending auditory interneurons in the neck connective, while stepwise increasing the insecticide concentration in the thoracic haemolymph. Furthermore, the insecticide dosages that showed clear effects on the auditory system were then also tested for systemic poisoning symptoms by injection experiments in 200 locusts.

## Materials and methods

### Animals

We used mature adult gregarious desert locusts *Schistocerca gregaria* (Forskål) of both sexes, that were purchased from a commercial insect breeder (BUGS-international GmbH, Germany). The fresh body weight of the animals was 1.7 ± 0.3 g for females and 1.3 ± 0.2 g for males. Locusts were housed in plastic terraria under crowded conditions with constant temperature of 25 ± 2 °C, 40–50% humidity, a 12:12 h light: dark cycle and were fed organic lettuce *ad libitum*. The experiments were carried out at room temperature (20–25 °C) and complied with the principles of Laboratory Animal Care and the animal protection laws and regulations of Germany and the European Union.

### Electrophysiology

**Fig. 1:**
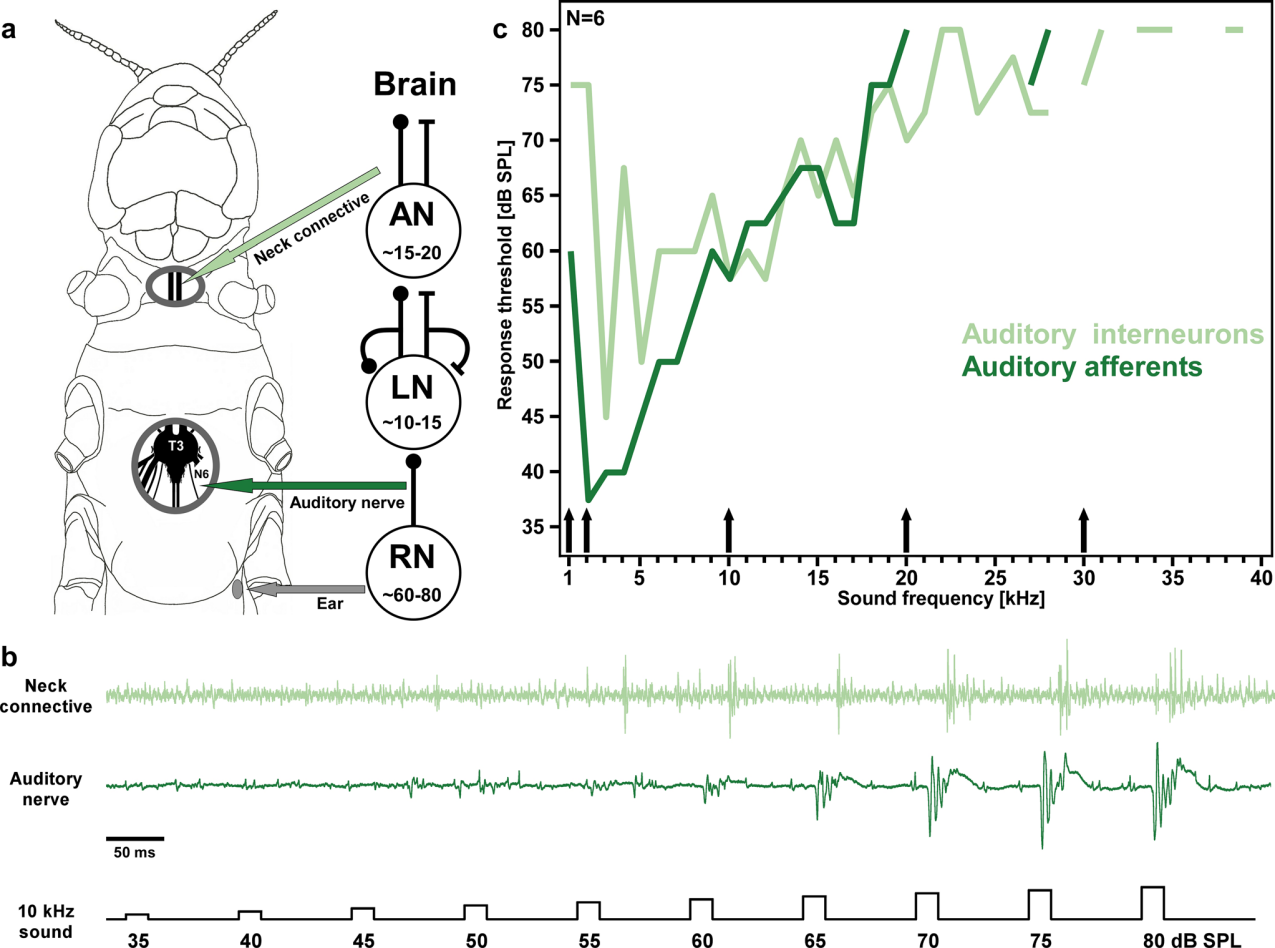
Experimental design. **a** Sketch of a locust head and thorax (left) with windows cut in the ventral cuticle to expose the neck connectives and the metathoracic ganglion with the auditory nerve for extracellular nerve recordings. Schematic wiring diagram of the locust auditory pathway (right; modified from Vogel and Ronacher 2007). Hook electrodes were placed at the auditory nerve (T3-N6; dark green arrow) to record the compound action potential response of the auditory receptor axons from the ear (grey arrow) and also at the neck connective (light green arrow) to record axonal spike responses of ascending auditory interneurons. Auditory pathway diagram illustrates the three processing stages from the ear to the brain including the respective number of auditory neurons (RN: receptor neuron, LN: local neuron, AN: ascending neuron). Filled circles indicate excitatory connections and t-shaped symbols indicates inhibitory connections. **b** Example of the extracellular nerve recordings. Bottom trace shows the acoustic stimulus, in this case 10 kHz sound pulses (20 ms each) with increasing amplitude from 35 to 80 dB SPL in 5 dB steps. Compound action potential responses from the sensory afferents in the auditory nerve (middle trace) and interneuron spikes in the neck connective recording (top trace). **c** Response threshold plot of auditory afferents (dark green) and auditory interneurons (light green) recordings show the hearing range of *S. gregaria* (median values, *N* = 6 animals). Based on these results, we chose five frequencies (1, 2, 10, 20, 30 kHz; indicated by black arrows) to cover the frequency range of locust hearing in the acoustic stimulation of the pharmacology experiments

In preparation for the recordings, both fore- and hindwings were removed, and the locust was fixed ventral side up on a plasticine-covered animal holder by restraining its legs and body with fitting metal clamps. A small window was cut in the metathoracic sternite and some trachea and fat tissue were carefully removed to expose the metathoracic ganglion with the auditory nerve (T3-N6) from the ear (Fig. 1a). A second window was cut in the soft cuticle between head and thorax to access the neck connectives. To prevent the nervous tissue from drying out, both openings were rinsed with insect ringer (NaCl 0,7%; KCl 0,02%; CaCl_2_ 0,02%; NaHCO_3_ 0,004%; pH 7.4).

Simultaneous extracellular recordings of the auditory nerve and the neck connective (Fig. 1b) were conducted with two double-hook electrodes (Schöneich et al. 2011). After hooking the nerve onto the electrode, the recording side was insulated with petroleum jelly to prevent short-circuiting by any fluid. The neck connectives were pinched with forceps anterior to the electrode position to eliminate spikes of descending interneurons in the recording. The electrode signals from auditory nerve and the neck connective were both amplified (1000x) and band-pass filtered (10–10000 Hz) with a differential amplifier (Model 1700, A-M Systems, Carlsborg, WA, USA) before digitally recorded and stored with 20 kHz sampling rate (A/D-converter Micro1401-4 and Spike2 v.10 software; CED, Cambridge, UK) for later off-line data analysis. Each electrophysiological recording for the pharmacological experiments took 3–4 h (see protocols for acoustic stimulation and pharmacological treatment). Since the signal amplitude of extracellular nerve recordings can slowly decay over time, we also measured a ringer control group. All together we recorded more than 50 locusts (*N* = 14 for each test group: imidacloprid, clothianidin, flupyradifurone and *N* = 11 for ringer control), but only analysed the data when the nerve recording was stable until the end of the experiment.

### Acoustic stimulation

For sound stimulation we used an external soundcard (fireface 400, RME, Audio AG, Germany) to drive a broadband loudspeaker (R2904, ScanSpeak, Vidbæk, Denmark). The speaker was placed perpendicular to the body axis (90° azimuth) at level with the animal (10 cm elevation over the table) and with 20 cm distance to the tympanum ipsilateral to the auditory nerve recording. Sound intensity was calibrated at the position of the animal for each frequency (1–40 kHz in 1 kHz steps) to 80 ± 2 dB SPL with a free field microphone (MK301, Microtech Gefell GmbH, Germany; sound calibrator type 4231 and measuring amplifier type 2610, Brüel & Kjær, Nærum, Denmark). The experiments were performed in a dark anechoic chamber lined with sound damping foam mats.

Trains of pure-tone sound pulses with 20 ms duration (1 ms rise and fall times; 80 ms pulse intervals) were computer generated using audio software (Audacity v.3.3, Audacity Team, https://audacityteam.org). The stimulation files were organized in blocks of 10 sound pulses with increasing intensity (from 35 to 80 dB SPL in steps of 5 dB; Fig. 1b) for each sound frequency. To evaluate the hearing range of *S. gregaria*, we tested sound frequencies from 1 to 40 kHz with 1 kHz steps (at least 17 stimulus repetitions) in *N* = 6 locusts. To keep the nerve recordings with pharmacological treatment within a reasonable time frame (3–4 h: 12 measurements of different drug concentration with 100–120 stimulus repetitions and 10 min break each), we limited the sound stimulation to 1, 5, 10, 20 and 30 kHz to cover the full hearing range of the animals (see arrows in Fig. 1c). For the data analysis the responses to all 5 different sound frequencies were pooled.

### Neuropharmacology

For each of the three investigated substances - imidacloprid, clothianidin and flupyradifurone (Sigma-Aldrich/Merck KGaA, Darmstadt, Germany) - the insecticide salt was dissolved and then further diluted in insect ringer to create for each substance 11 solutions with drug concentration increasing tenfold from 10^− 13^ to 10^− 3^ mol/l (0.1 pmol/l to 1 mmol/l). In the electrophysiology experiments an Eppendorf pipette was used to add 100 µl of the solution into the haemolymph of the thoracic body cavity housing the ear and the thoracic ganglia (bath application). After waiting 5–10 min for the drug to diffuse into the nervous tissue, the recording was started and then the sound stimulation file was played in loop-mode for about 10 min (100–120 loops). This protocol was repeated for all drug concentrations starting with insect ringer as the initial reference measurement and then subsequently increasing insecticide doses in tenfold steps from 10^− 13^ to 10^− 3^ mol/l, 100 µl each. In a control group the same protocol was applied with pure insect ringer instead of the insecticide solutions. Note that with this protocol of subsequently increasing insecticide concentrations, for each step there is an additive effect of 10% from the previous treatment.

For the injection experiments, the locusts were isolated individually in glass jars (400 ml, 85 mm diameter) with a perforated plastic lid and fed with fresh organic salad *ad libitum*. For each dosage (10^− 6^; 10^− 5^; 10^− 4^ and 10^− 3^ mol/l), five different treatment groups (*N* = 10 each) were analysed: control group (only handling without injection), ringer control group (injection of 100 µl insect ringer), imidacloprid, flupyradifurone and clothianidin group (injection of 100 µl of the respective insecticide solution). Injections into the haemolymph were applied with a 200 µl syringe (Hamilton Bonaduz AG, Switzerland) through the soft cuticle at the joint between hindleg coxa and metathorax. Each locust was checked 5 min, 2 h, 24 h, 48 h, 72 h and 96 h after the injection for visible symptoms of intoxication. Observed effects were categorized in symptom levels as follows: 0 - no obviously visible intoxication symptoms, 1 - muscle spasms, epileptic trembling of the whole body and legs, 2 - paralysis: often laying on the back, paralysed except for palp trembling in some individuals, 3 - dead, no movement or reaction detectable, stiffness and colour change of the body. For the injection experiments the experimenters did not know the treatment of the individual animals when observing and categorizing the intoxication symptoms. For the electrophysiological recordings the experimenter was not blinded.

### Data analysis

The electrophysiological data (for exemplary recording traces see Fig. 1b) was analysed using Spike2 (version 10, CED, Cambridge, UK) and R (version 4.1.2). To quantify the response in the auditory nerve recording, the signal was rectified before the waveform average was calculated. The R package ‘Bolstad2’ (version 1.0–29) (Curran 2013) was used to calculate integrals (area under curve). For comparison between locusts, the relative change to the signal at starting conditions (initial reference measurement with insect ringer) was calculated in percent. To quantify the spike responses in the neck connective recordings, a threshold was set at double the value of the baseline noise when no sound was played. Every spike above this threshold was counted and the average spike response (spikes within a 100 ms window after start of the sound pulse) was calculated after the spontaneous background activity (average spike number per 100 ms occurring during the initial 1 s of silence in the sound file) was subtracted. For comparison between locusts, spike response change in regard to the initial reference measurement was calculated in percent. Dose response curves were fitted with a 4-parameter logistic model (‘dr4pl’ R package version 2.0.0) (An et al. 2019) in two different ways. Once without restrains for comparison with the median data points of auditory responses and subsequently with a strictly forced starting point at 0% and a maximum of 100% effect magnitude to calculate effective concentrations (EC20, EC50, EC80) associated with 20, 50 and 80% response reduction.

Mean values are given with standard deviations (mean ± SD) for all normally distributed data. Median and interquartile range (IQR) were calculated for data sets that failed testing for normal distribution (Shapiro-Wilk normality test). Electrophysiological measurements of auditory nerve activity, auditory interneuron responses and background spike activity were compared respectively using the Kruskal-Wallis test with posthoc one-to-many Dunn´s test (‘PMCMRplus’ R package version 1.9.10, p-values were Bonferroni adjusted). To test for significant differences, each consecutive measurement of the insecticide treatment group was compared to the corresponding ringer control measurement. As a measure for effect size, we further calculated the eta squared (η^2^) that is given as a value between 0 and 1 (< 0.06: small effect, 0.06–0.14: moderate effect, > 0.14: large effect; ‘rstatix’ R package version 0.7.2). For the injection experiments, the Kruskal-Wallis test with Dunn’s multiple comparisons test (‘stats’ R package version 4.1.2; p-values were Bonferroni adjusted) was used to test for significant differences between multiple treatment groups. This test for non-parametric data sets is used because the symptom levels are quantitative but categorical. All figures were made using the ‘ggplot2’ R package (version 3.4.0) (Wickham 2016) and CanvasX-draw (Canvas GFX, Inc., USA).

## Results

**Fig. 2:**
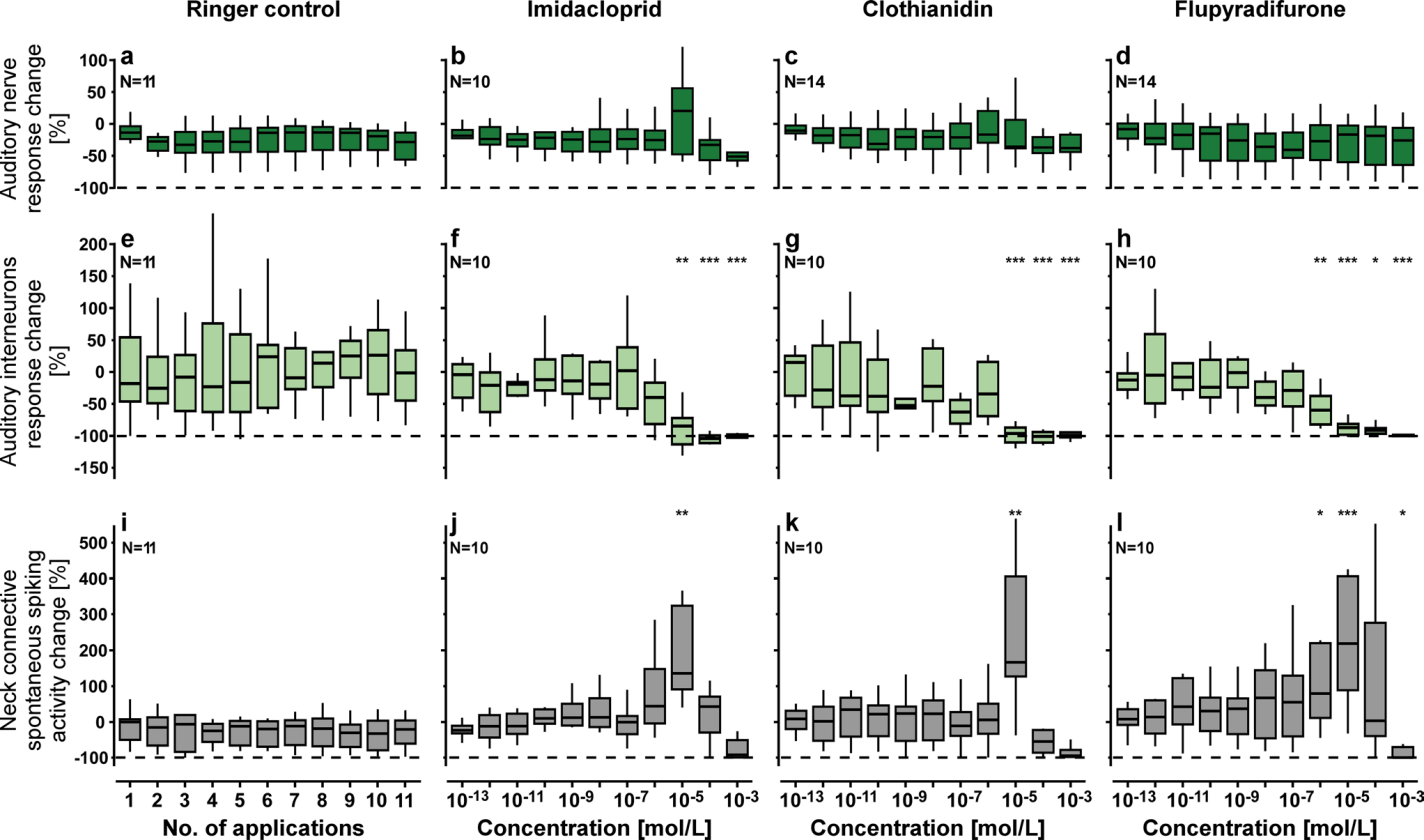
Insecticide impact on neuronal responses in the auditory pathway. Boxplots show the changes of auditory afferents responses (**a**-**d**), auditory ascending interneuron responses (**e-h**) and spontaneous spiking-activity in the neck connective (**i**-**l**) for ringer control (first column), imidacloprid (second column), clothianidin (third column) and flupyradifurone (fourth column) treatment. Neuronal activity changes with and without sound stimulation always refer to an initial reference measurement with pure saline for each individual animal (see methods for details). Boxplots show the median (horizontal line), interquartile range (box) and data distribution within 1.5 times of the IQR (whiskers). Asterisks indicate significant differences (*p* < 0.05*, *p* < 0.01**, *p* < 0.001***), always compared to the corresponding ringer control measurement (Kruskal-Wallis test with post-hoc one-to-many Dunn´s test; see methods for details). Note the spike response failure of auditory ascending interneurons for the 3 highest insecticide concentrations (10^− 5^-10^− 3^ mol/l, **f**-**h**). In contrast, the spontaneous spiking activity in the neck connective starts to increase with insecticide treatment of 10^− 6^ mol/l and vanishes at 10^− 3^ mol/l (**j**-**l**). The dashed lines indicate no response (-100% response change). N refers to the number of animals

**Fig. 3:**
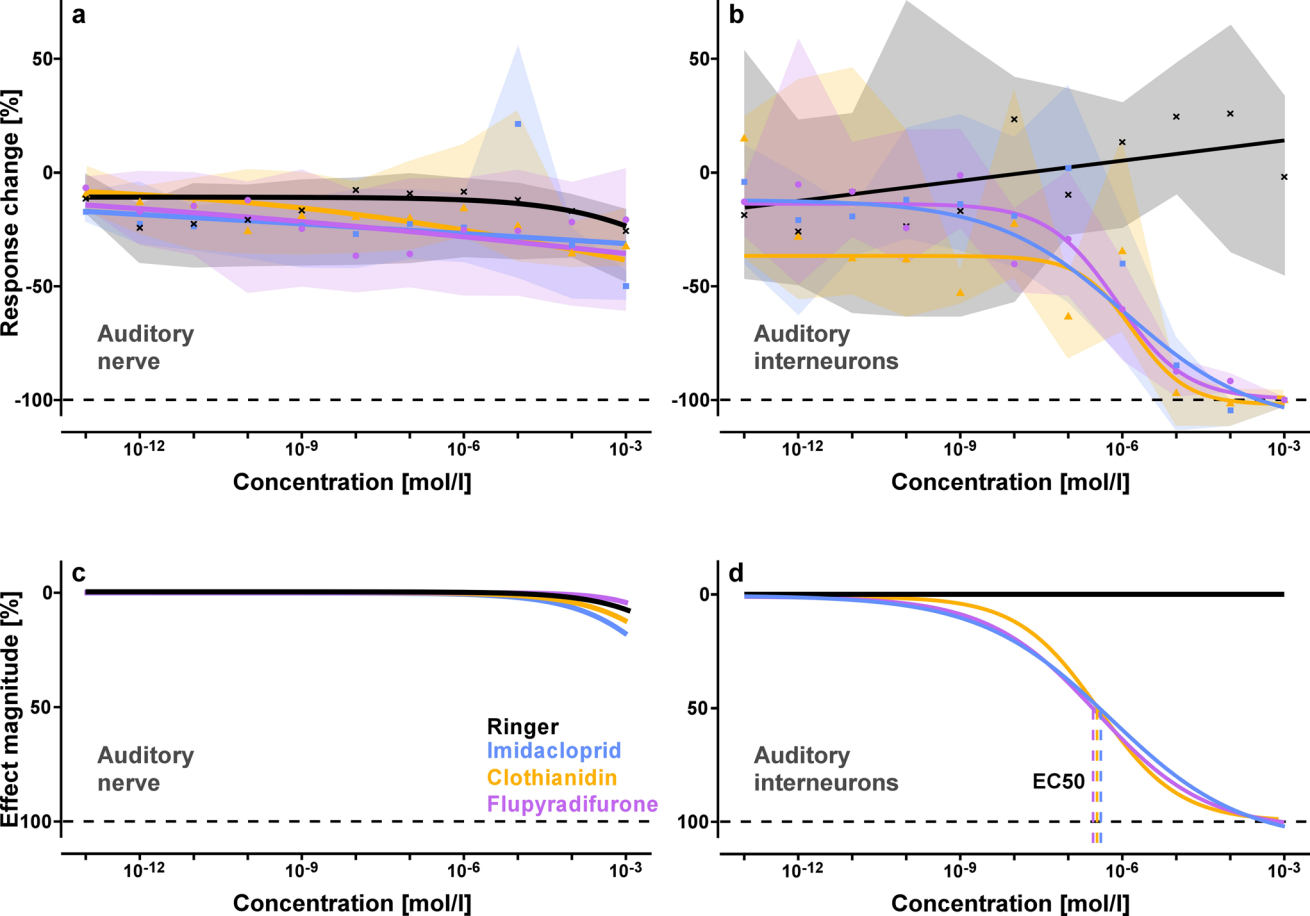
Dose-response curves fitted to the recording data using a 4-parameter logistic model (see methods) to compare the neural responses of auditory afferents (**a**, **c**) and ascending auditory interneurons (**b**, **d**) after treatment with different dosages of imidacloprid (blue lines and squares), clothianidin (orange lines and triangles) and flupyradifurone (purple lines and dots) with the ringer control (black lines and crosses). The horizontal dashed lines indicate no response (100% response reduction). **a**, **b** Unforced model fits are plotted with the corresponding median data points and interquartile range (shaded areas). **a** Responses of auditory afferents for the three insecticide treatments were similar to the ringer control. All 4 model curves show the same trend of slight amplitude decrease over the time of the experiment. **b** In comparison to the ringer control, there was a clear dose-dependent response reduction in auditory interneurons for all three insecticides. **c**, **d** To compare between the different treatments, the start of the model curves were force-fitted to 0 and for dose-dependent effects the EC50 (effective concentrations associated with 50% effect magnitude, indicated by vertical dotted lines) was calculated. **c** The forced model curves indicate no effect of the insecticide treatments on the auditory afferent responses. **d** For the response reduction in auditory interneurons, the EC50 was about 0.5 µmol/l for all three insecticides

To evaluate the impact of three different insecticides (imidacloprid, clothianidin and flupyradifurone) on neuronal processing of acoustic stimuli in locusts, we conducted extracellular nerve recordings at two different locations in the auditory pathway: from sensory afferents in the auditory nerve between the ear and metathoracic ganglion and simultaneously from ascending auditory interneurons in the neck connective between the thoracic and head ganglia (Fig. 1a, b). A sequence of sound pulses with 5 different frequencies within the hearing range of the locust (see black arrows in Fig. 1c) was repeatedly played for each measurement (see methods). For an initial reference measurement of the auditory responses, the thoracic body cavity was first filled with insect saline in each locust. Subsequently, 11 consecutive measurements after treatment with either pure insect saline (control group) or insect saline with increasing insecticide dosages (treatment groups) were analysed as response difference compared to the initial reference measurement in the same locust (Fig. 2). Our experiments revealed no obvious effects of the three different insecticides on the responses of the sensory cells in the auditory nerve, but clear dose-dependent suppression of spike responses in ascending auditory interneurons of the neck connective (Fig. 2). Semilogarithmic dose-response curves fitted with a 4-parameter logistic model emphasise the trends in the data (Fig. 3). We calculated unforced curves to first show how the models relate to the median data points (Fig. 3a, b) and then force-fitted them to start at 0 for slope comparison between the different treatment groups (Fig. 3c, d).

The compound action potential responses in the auditory nerve recordings of the ringer control experiments showed a small but consistent amplitude decrease over the time of the experiment (Fig. 2a), most likely due to a gradual decay of the signal amplitude during the recording (see supplementary figure S1). The same trend is also recognizable in the data sets for each of the three insecticide treatment groups (Fig. 2b-d). For 10^− 5^ mol/l imidacloprid treatment there was a notable increase of the activity in the auditory nerve recording, which was not statistically significant (*p* = 0.69, Fig. 2b). For 10^− 6^ mol/l treatment with clothianidin, we observed the same effect, less pronounced (Fig. 2c). However, this temporary increase in the auditory nerve activity was not stimulus-correlated as it was also present without sound stimulation (see supplementary figure S1). Specifically with these insecticide concentrations, we observed during some of the recordings a continuous trembling, twitching and spasm of all muscles in the locust’s body, which seems to have mechanically stimulated the ear. However, in all three insecticide treated groups the auditory nerve responses to the sound stimulation were not significantly different to the corresponding ringer controls (*p* > 0.5 each; Fig. 2b-d). Even with the highest insecticide concentration (10^− 3^ mol/l), the responses of the auditory afferents did not differ from the ringer control. The dose-response curves for the sensory afferents in the auditory nerve did not differ substantially from the ringer control, which showed a small but constant decrease in the recording signal over the relative long overall recording time (~ 4 h) of each experiment (Fig. 3a, c).

The spike responses in the neck connective recordings were variable between the individual locusts since in each experiment the hook electrode captured a different fraction of the 15–20 axons of ascending auditory interneurons in the locust. But for each individual recording the spike responses were stable over the time of the ringer control experiment (Fig. 2e). For the lower concentrations of all three insecticides the spike responses of auditory interneurons were constant with no significant differences to the ringer control (p-values from 10^− 13^ to 10^− 7^ mol/l: 0.99, 0.82, 0.75, 0.72, 0.25, 0.73, 0.07; Fig. 2f-h). At 10^− 6^ mol/l insecticide concentration, the spike responses of auditory interneurons differed significantly between treatment groups (*p* = 0.02). Responses were noticeably reduced for imidacloprid (*p* = 0.07, η^2^ = 0.17) and significantly reduced for flupyradifurone (*p* < 0.01, η^2^ = 0.43), with a moderate and not statistically significant reduction for clothianidin (*p* = 0.43, η^2^ = 0.09). For higher concentrations there was a strong reduction of the auditory interneuron responses with all three insecticide treatments being significantly different to the ringer control for 10^− 5^ mol/l (imidacloprid: *p* < 0.01, η^2^ = 0.65; clothianidin: *p* < 0.001, η^2^ = 0.74; flupyradifurone: *p* < 0.001, η^2^ = 0.68) and for 10^− 4^ mol/l (imidacloprid: *p* < 0.001, η^2^ = 0.71; clothianidin: *p* < 0.001, η^2^ = 0.74;flupyradifurone: *p* < 0.05, η^2^ = 0.60). At the highest tested concentration (10^− 3^ mol/l) auditory responses were completely absent in the neck connective recordings (imidacloprid: *p* < 0.001, η^2^ = 0.74; clothianidin: *p* < 0.001, η^2^ = 0.68; flupyradifurone: *p* < 0.001, η^2^ = 0.69). The dose-dependent inhibitory effects on the spike responses of ascending auditory interneurons between treatments become even more clear in the data-fitted model curves (Fig. 3b). The forced model curves (Fig. 3d) reveal that 50% response reduction in ascending auditory interneurons was reached at concentrations of about 0.4–0.5 µmol/l (10^− 7^-10^− 6^ mol/l) for all three insecticides (EC50: 0.35, 0.44 and 0.54 µmol/l for flupyradifurone, clothianidin and imidacloprid, respectively). A 20% response reduction was first reached with the imidacloprid treatment (EC20: 11, 14 and 40 nmol/l for imidacloprid, flupyradifurone and clothianidin, respectively), while 80% response reduction was first reached with clothianidin (EC80: 4.9, 8.8 and 25.7 µmol/l for clothianidin, flupyradifurone and imidacloprid, respectively). The auditory spike response in the neck connective was completely abolished with a drug concentration of 0.1 mmol/l (10^− 4^ mol/l) for all three insecticides, while the auditory afferents from the ear were unaffected by all tested insecticide concentrations.

For each treatment, we additionally analysed the spontaneous spiking activity of all ascending interneurons in the neck connective, which was constant during the ringer control experiments (Fig. 2i) and also for the low insecticide concentrations up to 10^− 7^ mol/l (Fig. 2j-l). In contrast to the strong reduction of sound-evoked spike responses in ascending auditory interneurons, the spontaneous spiking activity of non-auditory interneurons in the neck connective increased for the same insecticide treatments. Spontaneous spiking activity had its maximum at 10^− 5^ mol/l for all three insecticides, all differing significantly from the ringer control group (imidacloprid: *p* < 0.01, η^2^ = 0.74; clothianidin: *p* < 0.01, η^2^ = 0.55; flupyradifurone: *p* < 0.001, η^2^ = 0.50). All spiking activity of ascending interneurons in the neck connective, spontaneous spiking as well as sound responses, vanished almost completely at the highest tested concentration of 10^− 3^ mol/l for all three insecticides (Fig. 2f-h, j-l).

**Fig. 4:**
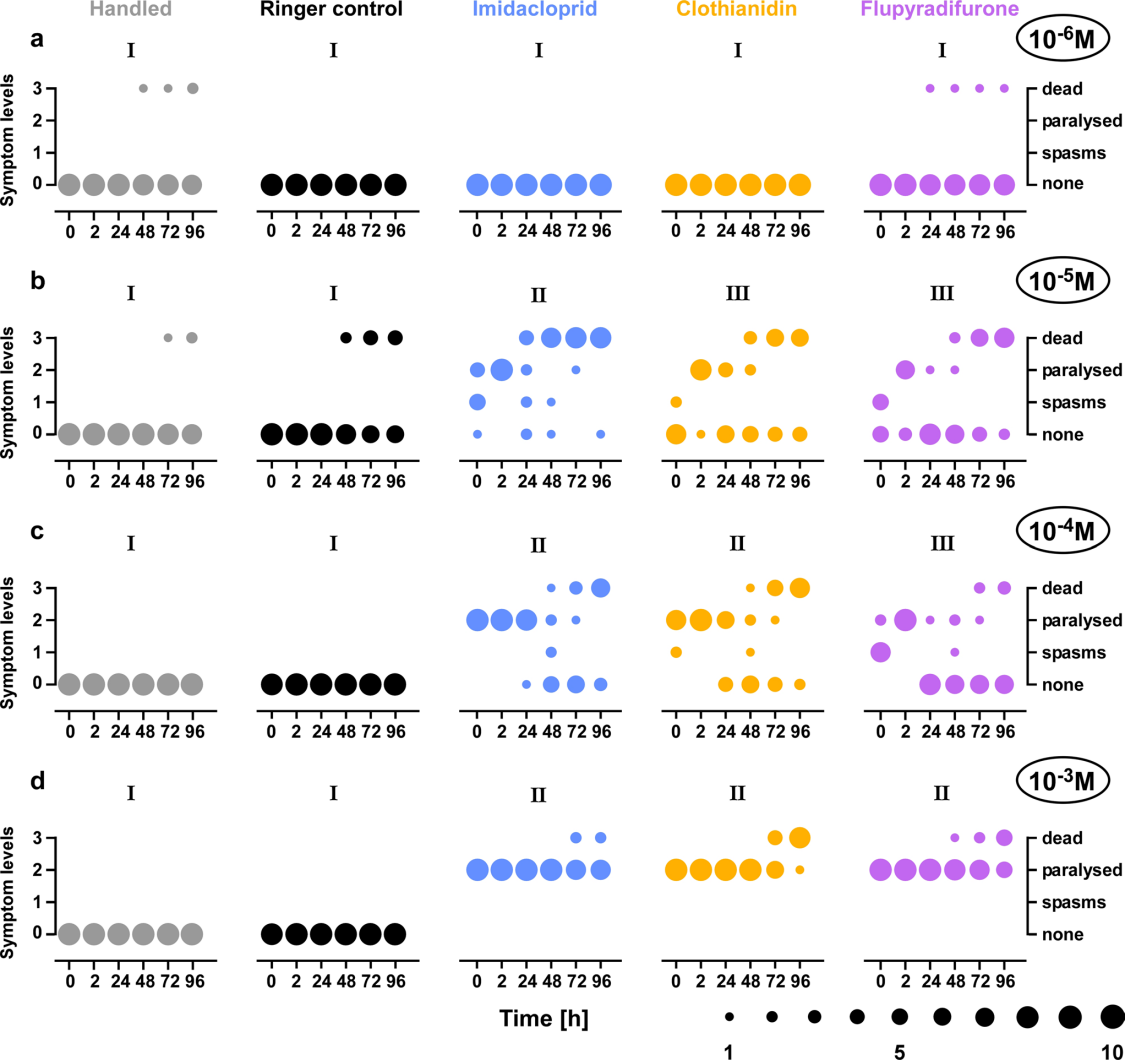
Symptoms of poisoning after injection. Control groups were only handled, ringer control groups with one injection of 100 µl ringer solution and insecticide groups with one injection of 100 µl of 10^− 6^ mol/l (**a**), 10^− 5^ mol/l (**b**), 10^− 4^ mol/l (**c**) or 10^− 3^ mol/l (**d**) of the respective insecticide. The circle size represents the number of animals in each category at the different times of observation (see bottom legend). Roman numerals indicate significant differences (*p* < 0.05): treatment groups with same letter are not different (Kruskal-Wallis test with posthoc Dunn’s comparison, see result section for individual p-values). **a** Injection of 100 µl of 10^− 6^ mol/l of the three insecticides (26 ng, 25 ng and 29 ng for imidacloprid, clothianidin and flupyradifurone, respectively) led to no obvious symptoms of poisoning. **b** Injection of 100 µl of 10^− 5^ mol/l insecticide solution (260 ng, 250 ng and 290 ng for imidacloprid, clothianidin and flupyradifurone, respectively) led to immediate poisoning effects including muscle spasms and paralysis, for all three insecticides. Some animals showed recovery from obvious symptoms. **c** Injection of 100 µl of 10^− 4^ mol/l of insecticides (2.6 µg, 2.5 µg and 2.9 µg for imidacloprid, clothianidin and flupyradifurone, respectively) led to strong immediate poisoning effects shown in all three insecticides including spasms and paralysis. Animals showed recovery from obvious symptoms. **d** Injection of 100 µl of 10^− 3^ mol/l of insecticides (26 µg, 25 µg and 29 µg for imidacloprid, clothianidin and flupyradifurone, respectively) led to quick and long-lasting paralysis with no instances of recovery for all three insecticides. **a-d** Note that the handled control and ringer control groups showed no side effect of experimental procedure

Based on the dose-dependent effects in the auditory network, injection experiments were conducted with insecticide dosages from 10^− 6^ up to 10^− 3^ mol/l, to evaluate behavioural symptoms of systemic poisoning. No obvious intoxication symptoms and therefore no significant differences could be observed between the two control groups and also after 100 µl injections of 10^− 6^ mol/l solution of the respective insecticides (Fig. 4a). During the entire observation time of 96 h, only one animal of the flupyradifurone group and two animals of the handling control group had died.

For all three insecticides, an injection of 100 µl of 10^− 5^ mol/l solution (Fig. 4b), led to immediate symptoms of poisoning including muscle spasms and paralysis in some animals (portion of animals with symptom levels 1–2 within 5 min after injection: 90, 50 and 20% for imidacloprid, flupyradifurone and clothianidin treatment, respectively) but not in any of the control group animals. Two hours after injection, most animals of the three insecticide groups were laying on the back, paralysed (symptom level 2). After 48 h most imidacloprid treated animals had died (80%), while in clothianidin and flupyradifurone treated animals, there was a split between animals that either had fully recovered (category 0: 50% for flupyradifurone, 70% for clothianidin) or had died (category 3: 30% for flupyradifurone, 20% for clothianidin). After 72 and 96 h most animals in the three insecticide treated groups had died (dead locusts after 96 h: 60, 80 and 90% clothianidin, flupyradifurone and imidacloprid treatment, respectively), while the majority of control group locusts were still alive (dead locusts after 96 h: 20 and 40% for handled control group and ringer injection, respectively). All three insecticide treatments led to significant differences compared to the handled control and ringer injection groups (ringer vs. flupyradifurone: *p* < 0.05; ringer vs. clothianidin: *p* < 0.01; all other *p* < 0.001), while the handled control and the ringer injection group did not differ significantly (*p* = 1). For imidacloprid injections we found the fastest and strongest effects with the lowest number of recoveries, which differs significantly from the other two insecticides (*p* < 0.001 each). There was no difference between flupyradifurone and clothianidin treatment (*p* = 1).

Injection with 100 µl of 10^− 4^ mol/l solution caused strong immediate poisoning effects, including muscle spasms and paralysis (symptom levels 1–2), for all three insecticides within 5 min (Fig. 4c). After 2 h, all insecticide treated animals were laying paralysed on the back (symptom level 2). After 24 h most imidacloprid treated animals were still paralysed, while clothianidin treated animals partly recovered and flupyradifurone treated animals mostly recovered (recovered animals: 10, 40 and 90% for imidacloprid, clothianidin and flupyradifurone, respectively). 48 h after injection a range of symptoms could be observed in all insecticide groups (symptom levels 0–3). After 72 and 96 h the animals were either dead or had recovered (dead animals after 96 h: 0, 0, 30, 70 and 80% for handled control, ringer injection, flupyradifurone, imidacloprid and clothianidin treatment, respectively). All three insecticide treatments with 10^− 4^ mol/l led to significant differences in comparison to the handled control and ringer control group (*p* < 0.001 each), while the handled control and the ringer injection group did not differ (*p* = 1). Among insecticide treatments, imidacloprid had the fastest and strongest effect with all animals being paralysed after 5 min. However, this differences in symptom levels did not differ significantly from the clothianidin treatment (*p* = 1). Flupyradifurone treatment resulted in the highest recovery rate and therefore also differs significantly from imidacloprid (*p* < 0.01) as well as clothianidin treatment (*p* < 0.05).

Injection with 100 µl of 10^− 3^ mol/l solution of the respective insecticides, evoked very strong immediate symptoms with all animals in these treatment groups being paralysed 5 min after injection (Fig. 4d, symptom level 2). In contrast to the other tested concentrations, this effect lasted for the entire 96 h of the experiment and there were no instances of recovery. Therefore, at the end of the experiment all insecticide treated animals were either still paralysed or had died (dead animals after 96 h: 0, 0, 20, 50 and 90% for handled control, ringer injection, imidacloprid, flupyradifurone and clothianidin treatment, respectively). All three insecticide treatments with 10^− 3^ mol/l led to significant differences in comparison to the handled control and ringer control group (*p* < 0.001 each), while the handled control and the ringer injection group did not differ (*p* = 1). No animal had died in either of the control groups. Clothianidin treatment resulted in most dead animals (90% after 96 h), while imidacloprid resulted in the least (20% after 96 h). Other than this, there were no overall differences (*p* = 1) between the 10^− 3^ mol/l insecticide treatments with all three leading to immediate and long-lasting paralysis.

## Discussion

Our data show that imidacloprid, clothianidin and flupyradifurone caused the same overall dose-depended effects at similar concentrations in adult locusts. For all three insecticides, systemic injection of 1 nmol (100 µl of 10^− 5^ mol/l) in the haemolymph was the lowest dose that caused obvious intoxication symptoms, which were ranging from appendage trembling and limb spasms to complete paralysis of the whole body (Fig. 4). Contrary to vertebrate motoneurons, which release acetylcholine directly at the muscle fibres, glutamate is the principle excitatory transmitter at the neuromuscular junctions in insects (Burrows 1996; Homberg 2002). Therefore, these visible intoxication symptoms indicate distinctive grades of malfunctioning in the premotor circuits that drive the motoneurons in the CNS. Similar disruption of motor functions had been reported for the same insecticides in the migratory locust *Locusta migratoria* (Parkinson et al. 2017) and also a variety of other insects (Tharp et al. 2000; Suchail et al. 2001; Williamson et al. 2014; Scheibli et al. 2023, 2024). After systemic injection of 10 nmol (100 µl of 10^− 4^ mol/l) each of the locusts in our experiments became paralysed within 2 h and was then laying on its back for 1–2 days before it either died or recovered. This ability to recover to apparently normal neural functions after a single injection may reflect the binding kinetics of the insecticides at the nAChRs in combination with a gradual metabolic breakdown and excretion (Bass et al. 2015). However, the chances for such recovery can be assumed to be substantially lower with chronically repeated exposure for insects on the fields (Tennekes 2010; Tennekes and Sánchez-Bayo 2013). An almost complete paralysis was observed in all locusts within less than 5 min after a single injection with 100 nmol (100 µl of 10^− 3^ mol/l) for each of the three tested insecticides. None of these locusts recovered within 96 h, but slight limb twitches occurred from time to time. If an insect remains paralysed like this for several days in the field, however, it would starve, desiccate or be eaten by predators (Hallmann et al. 2014; Wu et al. 2020b).

By activating postsynaptic AChRs (Crossthwaite et al. 2017; Taillebois et al. 2018; Matsuda et al. 2020), neonicotinoid insecticides typically cause long-lasting depolarization of the dendritic membrane. Opening of these cation channels in the cell membrane allows for sodium to enter and potassium to exit, but with an overall inward cation net flow that lowers the threshold for postsynaptic spike generation (Buckingham et al. 1997; Tan et al. 2007). Such lowered spiking threshold was reported in in the olfactory pathway of moths, where low dosages of clothianidin enhanced the sensitivity towards sex pheromones in central olfactory interneurons but did not affect the responses of the peripheral sensory cells (Rabhi et al. 2014, 2016). Similarly, neonicotinoids like imidacloprid act as an agonist to acetylcholine at the synapses between mechanosensory afferents from the cercus and wind-sensitive giant interneurons in the terminal ganglion of the cockroach (Buckingham et al. 1997; Yassine et al. 2013). In our experiments, this typical depolarizing (excitatory) effect on interneurons was reflected by a significant increase in the spontaneous spike activity in the neck connective recordings without auditory stimulation. The background spiking peaked after treatment with 1 nmol per locust (100 µl of 10^− 5^ mol/l), but then dropped drastically again for higher dosages (10 nmol) and was almost vanished after 100 nmol treatment for each of the three tested insecticides (Fig. 2i-l).

With acetylcholine as presumed excitatory transmitter at the synapses between auditory afferents and first-order auditory interneurons in the thoracic ganglia (Stumpner and von Helversen 2001), one may expect that treatment with neonicotinoids will enhance the synaptic gain and consequently increase spike generation in all interneurons along the auditory pathway. Surprisingly, in our experiments for the three tested insecticides the spike responses of ascending auditory interneurons became increasingly sparse for the same dosages that increased the spontaneous background-spiking of non-auditory interneurons in the neck connective (Fig. 2). This reflects that the processing of auditory information in the thoracic ganglia of the locust is more complex than a simple excitatory feedforward network. Contrary to crickets and bush crickets (Schöneich 2020; Cillov and Stumpner 2022), in locusts and grasshoppers the ascending auditory interneurons do not receive monosynaptic inputs from auditory afferents (Römer and Marquart 1984; Stumpner and Ronacher 1991; Vogel and Ronacher 2007). Instead, they receive excitatory and inhibitory inputs from intercalated local and bi-segmental auditory interneurons (Römer and Marquart 1984; Römer et al. 1988) and their spike responses critically depend on the balance between excitatory and inhibitory inputs (Römer and Rheinlaender 1983; Marquart 1985; Stumpner and Ronacher 1991). The reduction of spike responses in ascending interneurons may indicate that the neonicotinoids unevenly impact inhibitory and excitatory first-order auditory interneurons in a way that tips the balance towards inhibition within the network. This hypothesis could be directly tested in future studies by intracellular recording of excitatory and inhibitory postsynaptic potentials in the dendrites of local and ascending auditory interneurons in the thoracic ganglia. Even small changes in the balance between excitatory and inhibitory connection can drastically change the processing properties in small neural networks (Clemens et al. 2021), while greater imbalance can induce epileptic seizures or completely silence neural circuits (McCormick and Contreras 2001; Sohal and Rubenstein 2019).

Due to the experimental protocol with subsequently increasing insecticide dosages during the electrophysiological recordings, at each step an additive effect of 10% from the previous treatment with the lower concentration needs to be considered when comparing these results with the single injection experiments. Nevertheless, the neuronal processing in the auditory pathway already started to be affected after treatment with a much lower dosage than the occurrence of visible intoxication symptoms in the motoric behaviour. All three tested insecticides drastically reduced the ability of ascending interneurons to forward auditory information to the brain in a very similar dose-dependent fashion (EC50: 0.04–0.05 nmol per animal; Fig. 3). Comparison with the ringer control group shows that the spike responses of auditory afferents from the ear were not affected by the insecticide treatment. Even for the highest insecticide dosage tested (100 nmol = 100 µl of 10^− 3^ mol/l), the auditory response of the receptor neurons in the tympanic nerve did not change significantly whereas the spike responses of ascending auditory interneurons in the neck connective was largely supressed. In summary, our results support acetylcholine as an important excitatory neurotransmitter of the auditory pathway in the locust CNS, and particularly at the synaptic connection between auditory afferents and first order auditory interneurons in the thoracic ganglia (Stumpner and von Helversen 2001; Homberg 2002). Furthermore, our results are also in line with the current state of knowledge that the sensory neurons of tympanic ears in orthopteran insects are not under modulation by the central nervous system. In contrast to vertebrate ears (Elgoyhen and Katz 2012) and the antennal ears of mosquitoes (Andrés et al. 2016; Loh et al. 2023), there are no efferent neurons projecting into the Müller’s organ of the locust (Jacobs et al. 1999).

It is always very difficult to directly link effects on the cellular level observed in neurophysiological experiments under lab conditions to the exposure in the field. Most measurements of insecticide concentrations focused on nectar and pollen to estimate the potential harm for pollinators (Zioga et al. 2020). However, the concentrations in green leaves may be most relevant for locusts. The insecticide distribution in different parts of plants varies depending on plant species, method of application, and most importantly on the timing of measurement after the treatment (Stamm et al. 2015; Wu et al. 2020a). For flupyradifurone there is only few data for plant tissue, but up to about 50 µg/g was measured in watermelon (US EPA 2014). Imidacloprid concentrations in cotton leaves ranged from 1 to 50 ng/g after seed treatment (Jiang et al. 2018), but can be thousandfold higher shortly after spray application (Wu et al. 2019). This would be in the range of the physiologically relevant concentrations of our experiment (e.g. 100 µl of 10^− 6^ mol/l ≈ 25 ng active ingredient per animal), considering that each day an adult locust can easily consume the equivalent of its own body weight (1–2 g) in green plant material. Due to the vast number of factors that influence the actual insecticide levels, it is difficult to exactly pinpoint the field-realistic exposure. Even more so when considering accumulative intake of multiple pesticides over longer time periods (Kiljanek et al. 2017). Comparison between different insect species will be even more complex. The core elements of nAChR subunits are highly conserved among different insect groups (Jones et al. 2007), but different neonicotinoids differ in their binding properties depending on the distinct subunit compositions in specific receptor subtypes (Lu et al. 2022). Although this can potentially lead to huge differences in the dose-dependent effects across different species, a comparison between different studies is very difficult even for the same species due to the methodological differences and diverse ways results are reported (Nagloo et al. 2024). Most insecticide studies report lethal dosage (LD50) values that largely depend on the way of drug-application and observation time, which are not standardized. For example, in the honeybee *Apis melifera* the LD50 values for imidacloprid reported in the literature range from 0.9 to 6000 ng/g body mass (Nagloo et al. 2024). Our study, however, focused on sublethal effects and we found a similar impact on the postsynaptic processing of auditory information for the same concentrations of imidacloprid, clothianidin and flupyradifurone (EC50 6–10 ng/g body mass). We also observed the first visible signs of disruption in the motor behaviours in the injection experiments at the same dosage (170 ng/g body mass) for all three tested insecticides.

The paramount task for most adult insects is to avoid predators and to find a suitable mating partner, which often depends crucially on intraspecific communication between the sexes. A sublethal dosage of neonicotinoid insecticides can disrupt such communication systems on the sender side, e.g. by reducing calling behaviour and sex-pheromone production in moths (Navarro-Roldán and Gemeno 2017). On the receiver side it can impede mate finding by impacting the orientation behaviour and diminishing responsiveness towards sex pheromones in some wasps and moths (Rabhi et al. 2014, 2016; Tappert et al. 2017). In locusts it has been shown that neonicotinoids disrupt the visual processing underlying collision avoidance behaviour (Parkinson et al. 2017, 2020). Although *S. gregaria* do not acoustically communicate, many other orthopteran insects (e.g. grasshopper, crickets and bushcrickets) strongly rely on acoustic signals for mate finding and predator avoidance (Hoy et al. 1989; Marsat and Pollack 2012; Schöneich 2020). Even small disturbances of their hearing ability may result in negative effects on reproduction and survival.

In conclusion, our study demonstrates for the first time that sublethal intoxication with classical neonicotinoid insecticides like imidacloprid and clothianidin as well as the novel flupyradifurone severely impact the processing of acoustic information in the central auditory pathway of the locust. For all three tested insecticides, the spike responses of auditory interneurons in the neck connectives were supressed in a similar dose dependent manner while the spike responses of the sensory neurons in the tympanal nerve remained largely unaffected. Furthermore, relatively low sublethal insecticide dosages, which did not evoke any obvious intoxication symptoms like tremor or spasms, already caused a severe response reduction in the ascending auditory interneurons. Since the unaffected afferent neurons only have output synapses, our results indicate that the effects of the three tested insecticides are specifically on postsynaptic processing along the auditory pathway of the locust. Thus, this is a suitable model system for pharmacological studies to differentiate between generic effects on neuronal functions and postsynaptic processing in neural circuits. As the use of insecticides is not completely avoidable in modern agriculture, more research to understand the specific effects of sublethal intoxication in different organisms is crucial, which can ultimately help improving insecticide management to better protect ecosystems and biodiversity.

## Electronic supplementary material

Below is the link to the electronic supplementary material.

Supplementary Material 1

## Data Availability

The data generated and analysed during the study are available from the corresponding authors on reasonable request.
