# Supplementary material for: Flupyradifurone, imidacloprid and clothianidin disrupt the auditory processing in the locust CNS
Source: J Comp Physiol A Neuroethol Sens Neural Behav Physiol. 2025 Feb 13;211(3):311–25. doi: 10.1007/s00359-025-01735-8 (PMC12081486; doi:10.1007/s00359-025-01735-8)
Supplement: Supplementary file 1 — Supplementary Material 1 [file 359_2025_1735_MOESM1_ESM.docx]

**
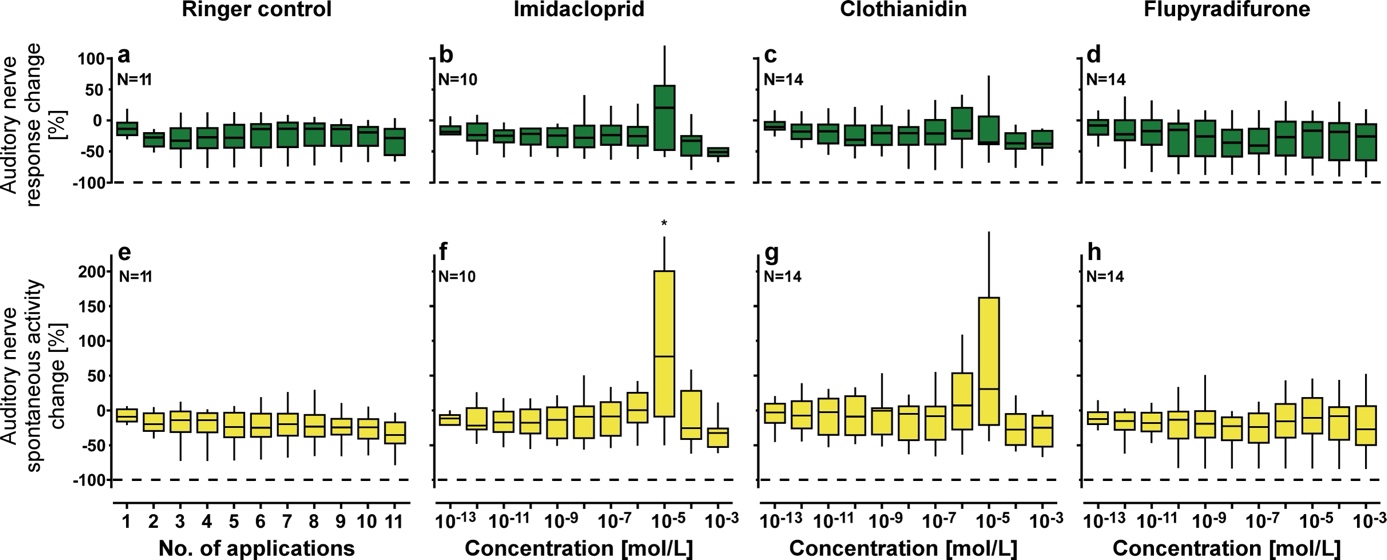
**

**Supplementary Figure S1.** Auditory afferents responses to sound stimulation (**a-d**) and 'spontaneous' activity of auditory afferents without sound stimulation (**e-h**) for ringer control (first column), imidacloprid (second column), clothianidin (third column) and flupyradifurone (fourth column) treatment. Auditory nerve responses to sound were calculated by subtracting the average 'spontaneous' nerve activity within a 100 ms time window before stimulation from the activity measured in 100 ms after the stimulus onset. The change in auditory nerve response and spontaneous activity always refers to an initial reference measurement at the beginning of the experiment for each individual animal (see methods for details). Note the higher activity measures from the auditory nerve in some of the animals with and also without sound stimulation after 10^-5^ mol/l imidacloprid and clothianidin treatments (notably larger IQRs). During these insecticide treatments, we also observed in the thorax of some animals a continuous trembling and muscle twitching, which most likely had enough mechanical impact on the ears to evoke sound-independent spiking in the auditory afferents. Ultimately, the auditory responses and spontaneous activity are both unaffected by insecticide treatments with higher concentrations (10^-4^ and 10^-3^ mol/l), which effectively paralyzed the motor system of the animals. Asterisk indicates the only significant difference (10^-5^ imidacloprid: p=0.03, η^2^=0.23) between insecticide treatment and the corresponding ringer control (Kruskal-Wallis test with post-hoc one-to-many Dunn´s test; see methods for details). Boxplots show the median (horizontal line), interquartile range (box) and data distribution within 1.5 times of the IQR (whiskers). The dashed lines indicate no response (-100% response change). N refers to the number of animals.
